# Nitrogen enrichment regulates straw decomposition and its associated microbial community in a double-rice cropping system

**DOI:** 10.1038/s41598-018-20293-5

**Published:** 2018-01-30

**Authors:** Tengfei Guo, Qian Zhang, Chao Ai, Guoqing Liang, Ping He, Wei Zhou

**Affiliations:** Ministry of Agriculture Key Laboratory of Plant Nutrition and Fertilizer, Institute of Agricultural Resources and Regional Planning, Chinese Academy of Agricultural Sciences, Zhongguancun No. 12, Beijing, 100081 PR China

## Abstract

Litter bag method was conducted to investigate the decomposition characteristics of rice straw (6000 kg ha^−1^) and its associated microbial community under different nitrogen (N) addition rates (0, 90, 180 and 270 kg N ha^−1^) under double-rice rotation. Generally, straw mass reduction and nutrient release of rice straw were faster in early stage of decomposition (0−14 days after decomposition), when easily-utilized carbohydrates and amines were the preferential substrates for involved decomposers. Straw-associated N-acetyl-glucosamidase and L-leucine aminopeptidase activities, which were higher under 180 and 270 kg N ha^−1^ addition, showed more activities in the early stage of decomposition. Gram-positive bacteria were the quantitatively predominant microorganisms, while fungi and actinomycetes played a key role in decomposing recalcitrant compounds in late decomposition stage. Straw residue at middle decomposition stage was associated with greater *cbhI* and *GH48* abundance and was followed by stronger β-glucosidase, β-cellobiohydrolase and β-xylosidase activities. Although enzyme activities and cellulolytic gene abundances were enhanced by 180 and 270 kg N ha^−1^ application, microbial communities and metabolic capability associated with rice straw were grouped by sampling time rather than specific fertilizer treatments. Thus, we recommended 180 kg N ha^−1^ application should be the economical rate for the current 6000 kg ha^−1^ rice straw returning.

## Introduction

Crop straw represents a major source of carbon (C) in agroecosystems^1^. Its decomposition involves the mineralization and transformation of photosynthesis products into stable soil organic matter, which plays the main role in resource recycling and soil nutrient transformation^2^. Rice is one of the most important agronomic plants in the world, and over 135 million ha of land is used for rice cultivation^3^. Approximately, 34.4 Tg year^−1^ of crop residue is produced worldwide, which may lead to sequestration of 200 Tg C year^−1 ^^4^. The increase in paddy soil organic C sequestration favors agricultural sustainable development and reduces global warming impact^2^. Given the importance of straw decomposition in soil C sequestration, there are interests in elucidating the dynamic changes of straw mass and populations of actors (i.e. enzymes, earthworms and fungi) during the course of straw decomposition at all time^5^.

Biochemical decomposition of rice straw is a sequential process that initially involves the loss of less recalcitrant components (i.e. oligosaccharides, organic acids, hemicellulose and cellulose) followed by the degradation of the remaining highly recalcitrant compounds (i.e. lignin or suberin). Straw mass changes during the course of its transformation and so does the activity and composition of the associated microbial communities^6^. For example, bacteria dominate in the initial phases, while fungi or actinomycete show higher abundance in the later stages of straw decomposition^7,8^. Hydrolytic enzymes are responsible for the acquisition of C, nitrogen (N) and phosphorus (P) to support primary metabolism and oxidative enzymes degrade recalcitrant compounds such as lignin in metabolic acquisition of nutrients^9^. These changes reflect the dynamically catabolic capabilities during straw decomposition process. However, enzyme activities and microbial community structure that engaged in straw decomposition vary with various environmental factors such as temperature, soil moisture and nutrient availability. And they arise under complex alteration of the aerobic and anaerobic environment during the rice-planting period^10^.

From a long-term perspective, straw incorporation is a beneficial practice to alleviate soil degradation, maintain soil fertility, and promote crop yields in intensive agricultural systems^11^. However, straw returning in a short period always increases soil N immobilization and mineralization, thereby causing N deficiency and yield decline^12^. Thus, simultaneous N fertilizer application with straw returning has been widely suggested and accepted. However, interactions between straw nutrient release (e.g. C) and N availability are poorly understood. The enzymatic reactions which catalyzes the chemical breakdown of plant residues, as well as the growth kinetics of microorganisms secreting those digestive enzymes, might be accelerated by the alteration of N fertilizer rates. Although numerous studies have demonstrated that C decomposition rate is affected by the biochemical composition of straw, such as contents of C, N, cellulose and lignin, and the ratios of C/N and lignin/N^13^, specific differences of rice straw under application of different N fertilizer rates have not been studied systematically. The varying dynamics of straw-associated enzyme activities, microbial community structure and metabolic abilities throughout different stages of decomposition require further research.

Therefore, a systematic study under double-rice rotation was conducted to determine the effects of N fertilizer rate on (1) rice straw decomposition rate, nutrient release pattern and representative enzyme activities involved in straw decomposition, (2) structure and metabolic activity of the rice straw-associated microbial community and (3) straw-associated cellulolytic fungi (*cbhI*) and actinobacteria (*GH48*) gene abundances. The results were expected to provide scientific information to support decisions on optimizing fertilizer N rates with a straw returning strategy in double-rice cropping system.

## Results

### Straw decomposition rate and nutrient release

**Figure 1:**
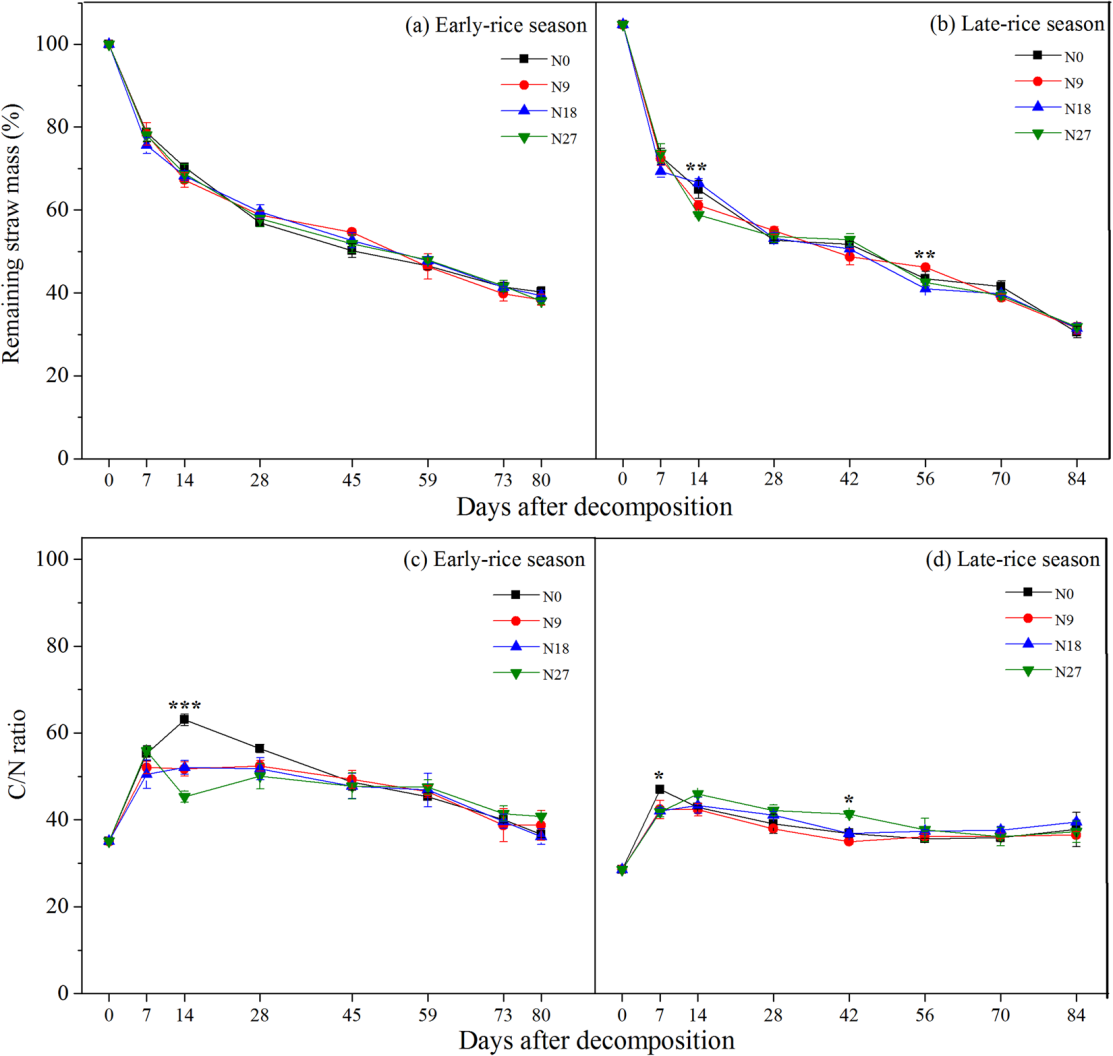
Temporal dynamics of the remaining straw mass and rates of straw decomposition (C/N ratio) in the early-rice and late-rice seasons.

Soil moisture and temperature dynamics were listed in this double-rice experiment (Supplementary Figure S1). In general, the loss of straw mass was faster in the early stage of decomposition (d0–d28) than in the late stage, especially in early-rice season. By the end of the harvest period, about 60–70% of returned straw was decomposed. The temporal dynamics of the remaining straw mass were approximately consistent between two seasons (Fig. 1a and b), whereas the mass proportion of remaining rice straw decreased more rapidly in late-rice season (Fig. 1b). At the same time, differences in the remaining straw mass under different fertilizer treatments varied significantly at d14 and d56 after decomposition in late-rice season (*P* < 0.05), where straw residual mass was the highest under the N18 treatment at d14 and lower at d56 (Fig. 1b). The cumulative rate of C and N release during straw decomposition in the first week was significantly higher in late-rice season than in early-rice season (Supplementary Figure S2a–d). The release of N during d0–d14 in both seasons was faster than the simultaneous release of C, resulting in the initial increase of the C/N ratio (Fig. 1c and d). The application of N fertilizer, especially with higher amounts (N18 and N27), delayed the N release of straw with relatively lower C/N ratio at d7 in early-rice season (Fig. 1d). In comparison, straw P and K were rapidly released within a week and were significantly accelerated by 180 kg N ha^−1^ addition at d14 (Supplementary Figure S2g and h), except for the release of P in early-rice season of which the cumulative release rate of P was only about 50% by d14 after burial in the field (Supplementary Figure S2e).

The decomposition rates for different fertilizer treatments in two seasons were well described by Olson’s (1963)^14^ exponential model, with *r*^2^ values ranging from 0.8896 to 0.9461 (Table 1). The decomposition rate (per year) was generally slower in early-rice season than in late-rice season. In early-rice season, straw under the N18 treatment showed the slowest decomposition rate, taking more than 1.43 years to decompose 95% of the applied rice straw, and N9 showed the highest decomposition rate. In late-rice season, straw under the N27 treatment showed the slowest decomposition rate and the time taken to reach 95% decomposition was the longest (at about 1.23 years). In contrast, N18 treatment showed the highest decomposition rate. However, these differences were not significant in both early-rice and late-rice seasons. The ABT analysis confirmed the strong effect of different seasons on decomposition rate and time for 50% and 95% decomposition, which was followed by soil temperature and moisture (Supplementary Table S1 and Figure S1). Fertilizer treatments showed a statistically less significant influence than environmental factors on straw decomposition parameters.

**Table 1:** Regression equations based on the Olson (1963)^14^ exponential model to describe litter decomposition under different fertilizer treatments.

Treatments	Regression equation	Correlation coefficient (*r*^2^)	Decomposition rate (year^−1^)	Time for 50% decomposition (year)	Time for 95% decomposition (year)
Early-rice season					
N0	*Y* = 85.456e^−3.753*t*^	0.9307	3.753	0.784	1.398
N9	*Y* = 86.180e^−3.858*t*^	0.9461	3.858	0.765	1.362
N18	*Y* = 85.043e^−3.673*t*^	0.9400	3.673	0.800	1.427
N27	*Y* = 85.800e^−3.786*t*^	0.9415	3.786	0.779	1.387
Late-rice season					
N0	*Y* = 80.662e^−4.304*t*^	0.9061	4.304	0.671	1.206
N9	*Y* = 79.422e^−4.232*t*^	0.9034	4.232	0.678	1.222
N18	*Y* = 79.668e^−4.321*t*^	0.9102	4.321	0.665	1.198
N27	*Y* = 78.980e^−4.208*t*^	0.8896	4.208	0.681	1.228

### Dynamics of enzyme activities during straw decomposition process

The dynamics of extracellular enzyme activities involved in C and N cycling under different fertilizer treatments during the straw decomposition process were assessed and presented in Supplementary Figure S3a–f. In general, C-related enzymes β-glucosidase, β-cellobiosidase and β-xylosidaseactivities increased with the decomposition process (lower in the early stage and higher in the late stage; Supplementary Figure S3a–c). In contrast, the N-related enzymes N-acetyl-glucosamidase and L-leucine aminopeptidase had higher activities in the early decomposition stage (d0-d28), and then decreased and stabilized with further straw decomposition (Supplementary Figure S3g–j). The enzymes phenol oxidase and peroxidase, which play an important role in straw lignin decomposition, only showed higher activities in the late decomposition stage with the highest phenol oxidase activity at d73 (or d70) and the highest peroxidase activity at d59 (or d56), respectively (Supplementary Figure S3k–n). Moreover, the N18 and N27 treatments had greater effects on C- and N-related enzyme activities than the N0 and N9 treatments (except phenol oxidase) in late-rice season. In general, N27 treatment had a greater effect than N18 treatment on β-xylosidase, N-acetyl-glucosamidase, L-leucine aminopeptidase and peroxidase activities in early-rice season, but N18 treatment had a greater effect than the N27 treatment on enzymes such as β-glucosidase, β-cellobiosidase, L-leucine aminopeptidase, phenol oxidase and peroxidase activities in late-rice season. The ABT analysis revealed clearly different enzyme activities under different decomposition stages with greatest contribution of sampling time to the variability of enzyme activities (Supplementary Table S2). Different N fertilizer rates were the second most important factor affecting C- and N-related enzyme activities, followed by soil temperature. Soil temperature had a comparatively higher effect on oxidase activities than hydrolase activities (Supplementary Table S2).

### PLFA analysis of microorganisms associated with straw decomposition

**Figure 2:**
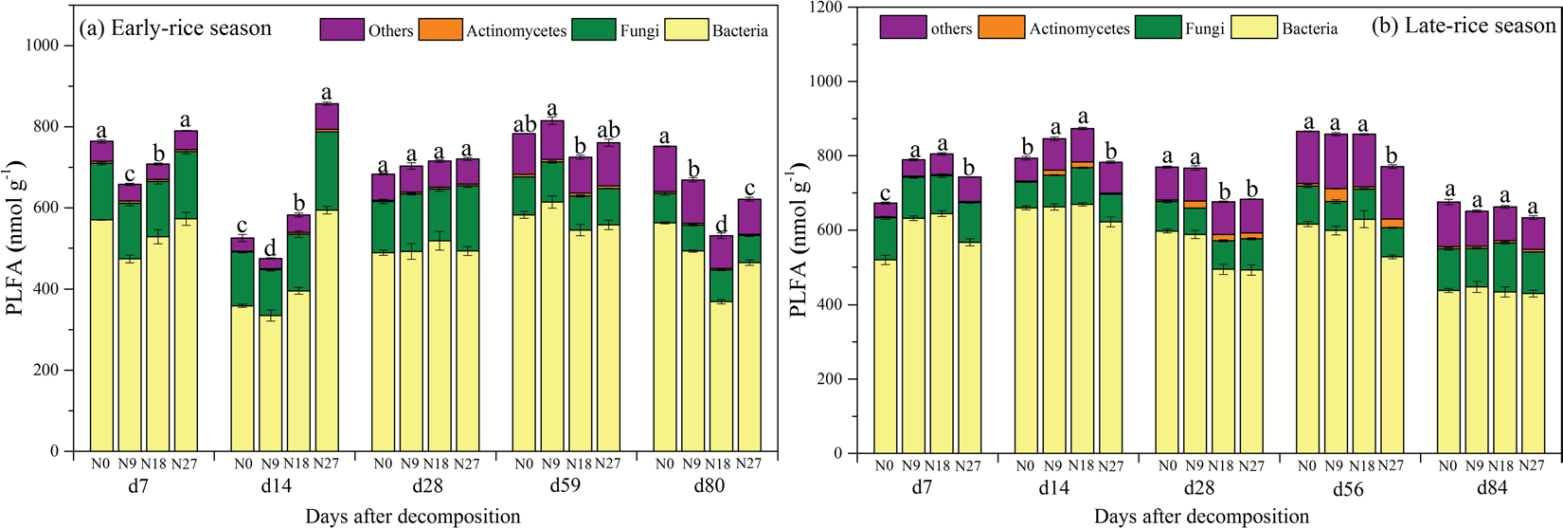
Microbial biomass as measured by phospholipid fatty acid (PLFA) analysis in the early-rice and late-rice seasons. Data are means of three replicates for each of the three groups of microorganisms (bacteria, fungi and actinomycetes). “Others” refers to PLFAs that are not strictly associated with three groups.

PLFA profiles were employed to estimate the microbial biomass, and the results are shown in Fig. 2. Total PLFAs and bacterial PLFA abundance generally dropped at d14, increased at d28 and became stable at d80 in early-rice season (Fig. 2a). Total PLFA abundance under N27 treatment was the highest among the four fertilizer treatments at the early decomposition stage (d0-d28). Fungal PLFA abundance was higher during the early decomposition stage (d7-d28) than in late stage (d59 and d80) of early-rice season, which was also higher under N18 and N27 treatments than the other treatments in early decomposition stage (Fig. 2a). Meanwhile, total PLFA abundance of N27 treatment was highest at d14, dropped to 84.08% at d28 and further dropped to 72.51% at d80 in early-rice season. As for the microbial composition index, G+/G− ratio started to increase at d28 and showed reduction at d80 (Supplementary Figure S4a). Fungi/bacteria ratios showed an opposite trend with G+/G− ratio which was higher in early decomposition stage and lower in late decomposition stage (Supplementary Figure S4b). Microbial community associated with straw residue under N18 treatment showed significantly higher fungi/bacteria ratio and lower G+/G− ratio than the other fertilizer treatments at the harvest period of early-rice season (Supplementary Figure S4a and b).

It is predicted that microbial biomass is high during the early stage of straw decomposition and then declines along with the decrease of available carbon sources. Such a trend was generally observed in late-rice season across four fertilizer treatments except at d56 after decomposition (Fig. 2b). Fungal PLFA biomass was higher at d7 and d84 than that at the other sampling time, which resulted in their relatively higher fungi/bacteria ratios (Supplementary Figure S4d). At the same time, straw-associated microbial community under N0, N9 treatments and N18, N27 treatments showed different pattern of G+/G− ratio which were gradually reduced along with straw decomposing process under N18 and N27 treatments except at d56 (Supplementary Figure S4c). However, G+/G− ratios of straw without N fertilizer addition or with least N fertilizer amount (N0 and N9) increased at d28 and subsequently decreased (Supplementary Figure S4c). In spite of the variable pattern of microbial community composition in early-rice and late-rice seasons, N18 treatment still held significantly higher fungi/bacteria ratio (Supplementary Figure S4d) and lower G+/G− ratio (Supplementary Figure S4c) than the other fertilizer treatments at the harvest period of late-rice season.

**Figure 3:**
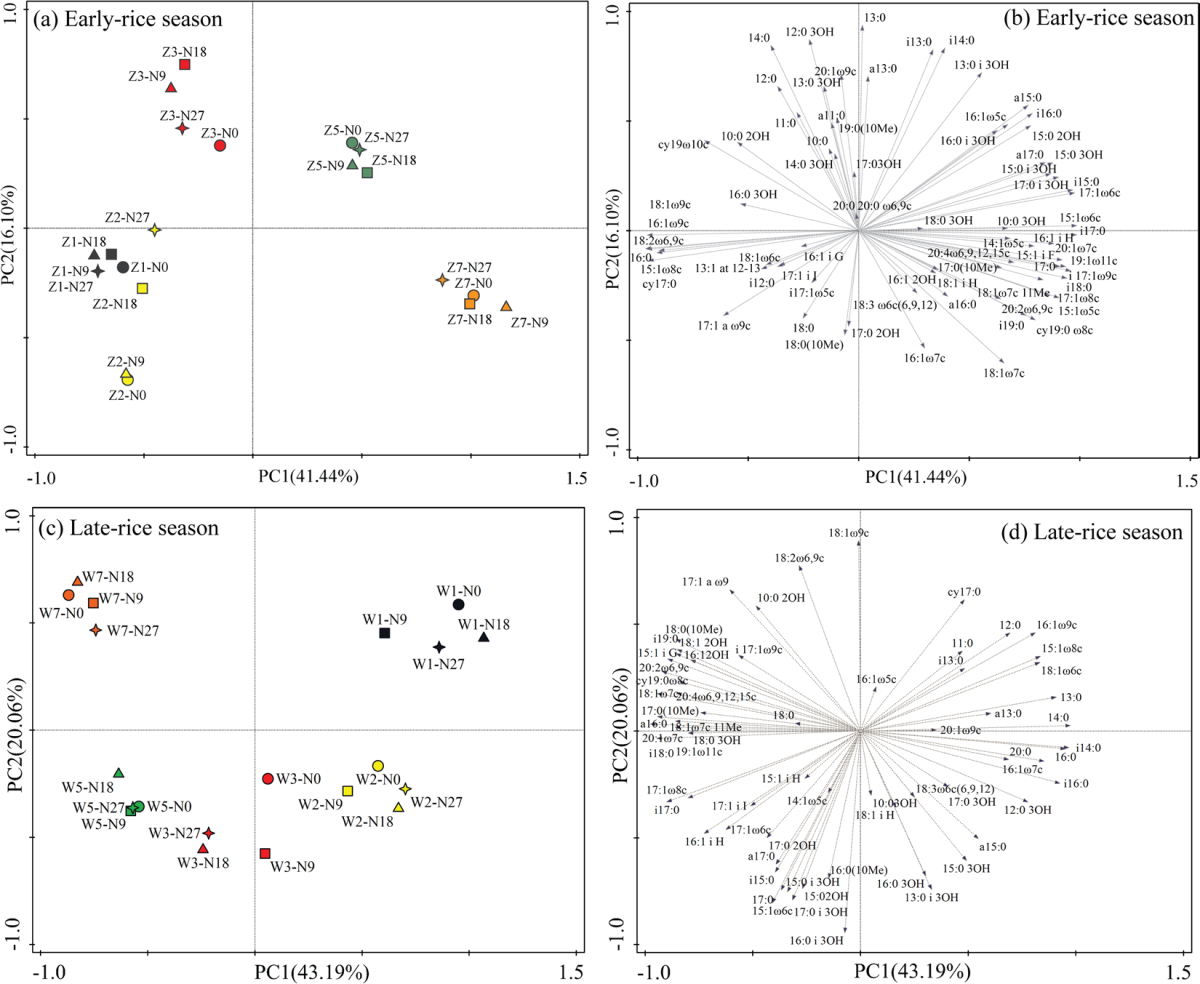
Principal component analysis (PCA) of straw-decomposing microbial communities from residue samples under different fertilizer treatments based on the individual PLFA (mol %). Z1-Z7 and W1-W7 referred to the sampling time as d7, d14, d28, d56 and d80 (d84) in early-rice season and late-rice season, respectively.

Furthermore, PCA (principal component analysis), used to compare the effects of N fertilizer rates and sampling time on straw-associated microbial community composition, was presented in Fig. 3. PC1 and PC2 accounted for about 40% and 20% in both seasons. The bioplot showed that microbial community composition associated with straw residue was clearly clustered by sampling time instead of fertilizer treatments in both rice seasons. In early-rice season, the proportions of monounsaturated fatty acids (15:1ω8c, 16:1ω9c, 18:1ω6c, 18:1ω9c), polyunsaturated fatty acids (18:2ω6, 9c), cyclopropane fatty acids (cy17:0) which represent fungi increased in the early decomposition stage (d7 and d14) (Fig. 3b). The saturated fatty acids (11:0, 12:0, 13:0, 14:0, i13:0, a13:0, i14:0, a15:0, i15:0, i16:0 and a17:0) (biomarkers of G+) and hydroxy fatty acid (10:0 2OH, 12:0 2OH, 13:0 2OH, 14:0 3OH, 15:0 2OH, 15:0 3OH, 13:0 i 3OH and 16:0 i 3OH) predominant in the middle stage of straw decomposition. The polyunsaturated fatty acids (14:1ω5c, 15:1ω5c, 16:1ω7c, 17:1ω8c, 18:1ω7c, 19:1ω11c, 20:2ω6, 9c, 18:3ω6c (6, 9, 12), and 20:4ω6, 9, 12, 15c) and cyclopropane fatty (cy19:0ω8c), most of which were biomarkers of G-, showed relatively importance than the other microbial groups in the late decomposition stage (Fig. 3).

Meanwhile, the monounsaturated fatty acids (15:1ω8c, 16:1ω5c, 16:1ω9c and 18:1ω6c) and cyclopropane fatty acids (cy17:0) took relatively important position in the early decomposition stage of late-rice season (Fig. 3d). Along with the decomposition process, the proportion of saturated fatty acids (15:0, 16:0, 17:0, i14:0, a15:0, i15:0, i16:0, i17:0, a17:0 and 20:0) (biomarkers of G+) and hydroxyl fatty acid (10:0 3OH, 12:0 3OH, 15:0 2OH, 15:0 3OH, 16:0 3OH, 17:0 2OH, 17:0 3OH, 13:0 i 3OH, 15:0 i 3OH and 17:0 i 3OH) increased at d14-d56. While at the harvest period, polyunsaturated fatty acids (18:1ω9c, 18:2ω6,9c, 19:1ω11c, 20:1ω7c, 20:2ω6, 9c and 20:4ω6, 9, 12, 15c), cyclopropane fatty (cy19:0ω8c) and methyl branched fatty acids (17:0 (10Me) and 18:0(10Me)), most of which were fungi and actinomycetes biomarkers, increased along PC2. Therefore, the results of PCA supported the general conclusion drawn from the ABT analysis that the effects of sampling time were larger than those of fertilizer treatment on decomposing microbial communities (Supplementary Table S3).

### CLPP analysis of microbial communities associated with rice straw

**Figure 4:**
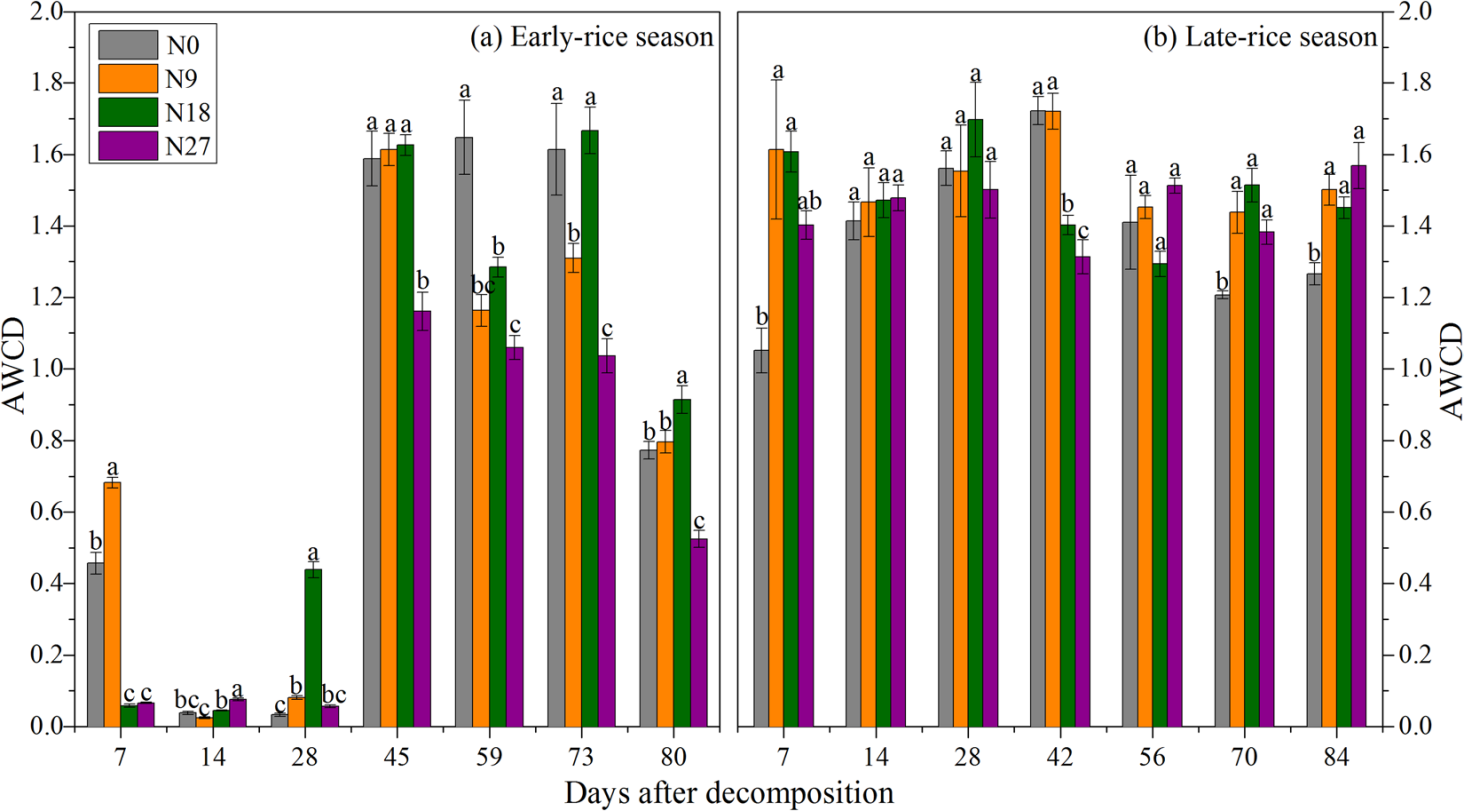
Metabolic activities of bacterial communities (AWCD, average well color development) associated with straw decomposition as estimated using the Biolog EcoPlate analysis. The vertical error bars represent standard error (s.e. = standard derivation/sqrt(n), *n* = 3) and are followed by a lowercase letter indicating a significant difference among fertilizer treatment within each sampling time according to Fisher’s least significant difference test (*P* < 0.05).

Metabolic activity of straw-associated microorganisms was examined using the Biolog EcoPlate system, a technique known as CLPP analysis^11^. The ability to utilize 31 carbon sources was assessed in triplicate, and the data were calculated as AWCD. As shown in Fig. 4a, AWCD scores for different fertilizer treatments were generally relatively higher in d45-d80 and lower in early stage of straw decomposition (d7-d28) in early-rice season. Compared with N0, N9 and N18 treatments, returning straw under N27 treatment showed relatively lower metabolic activity in the late decomposition stage (d28-d80). Metabolic activity of bacterial community associated with straw residue under N18 treatment was relatively higher at d28, d45, d73 and d80 than the other treatments. Notably, this trend did not hold in late-rice season which did not vary that much through decomposition process (Fig. 4b). Generally, the metabolic activities of straw residue under different N rate applications were higher in early and middle stage (d7-d42), especially N9 and N18 treatments, than in late decomposition stage (d56-d84). Compared with N0 treatment, N fertilizer addition significantly increased the microbial metabolic activities of straw residue at d7, d70, d84 in late-rice season (Fig. 4b).

**Figure 5:**
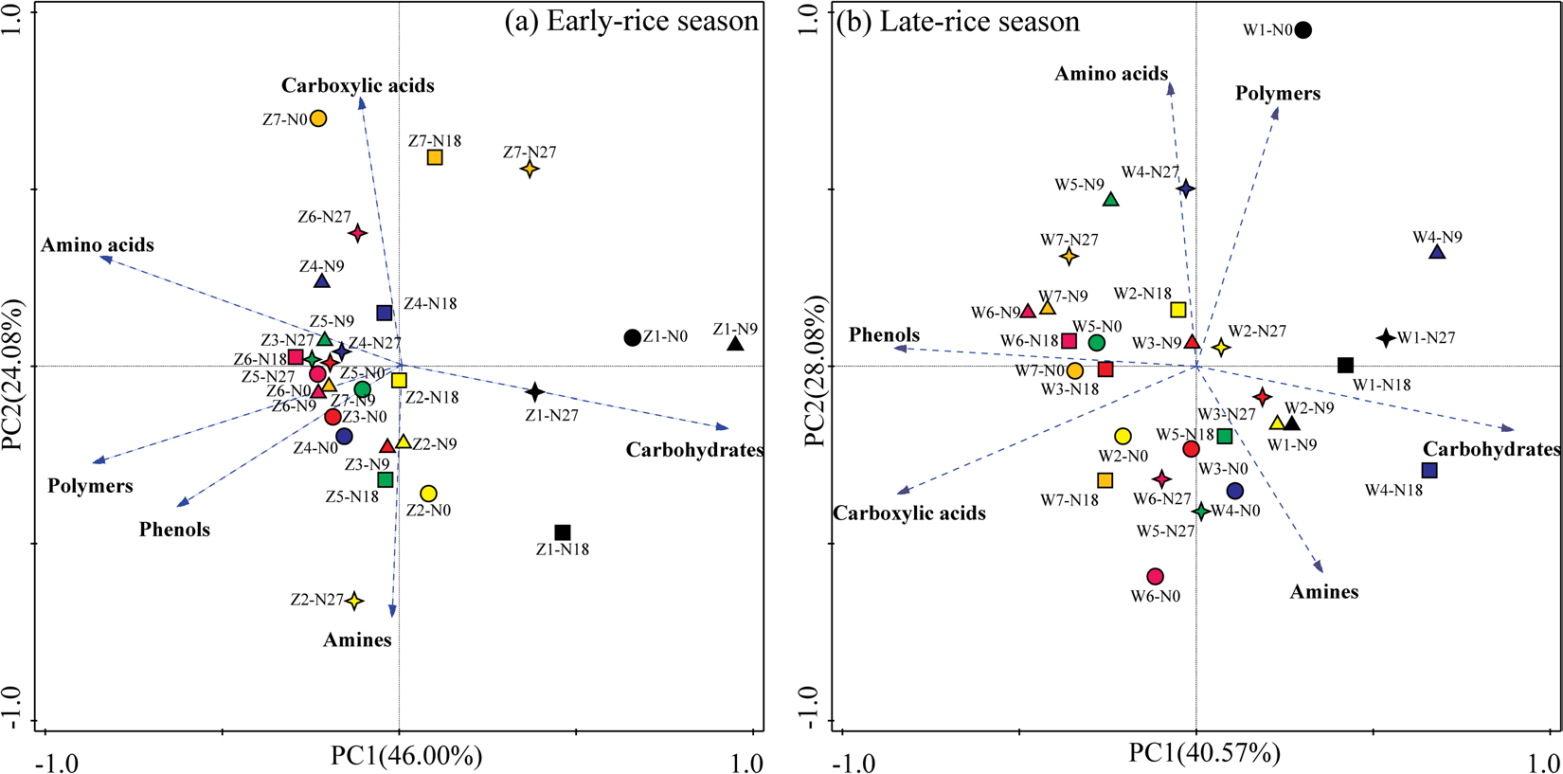
Principal component analysis (PCA) of straw-decomposing microbial communities of different residue samples under different fertilizer treatments based on the average well color development (AWCD) of carbon sources. Z1-Z7 and W1-W7 referred to the seven sampling time at d7, d14, d28, d45 (d42), d59 (d56),  d73 (d70) and d80 (d84) in early-rice season and late-rice season, respectively.

PCA results showed that C sources that straw decomposing microorganisms preferred were carbohydrates and amines during early decomposing stage (d7-d28) in both seasons (Fig. 5). C sources of carboxylic acids and amino acids in early-rice season and carboxylic acids and phenols in late-rice season were dominant substrate for microbial community associated with straw residues during late decomposition stage. However, there was no obvious cluster of specific fertilizer treatment in both seasons. When the functional diversity was calculated in the form of diversity indices, multivariate analysis revealed significant effects of different seasons and sampling times on AWCD values, *U*, *D* and *H* indices (Supplementary Table S3). Also, soil moisture and temperature influenced *D* and *H* indices.

### Straw decomposition-associated genes *cbhI* and *GH48* gene abundance

**Figure 6:**
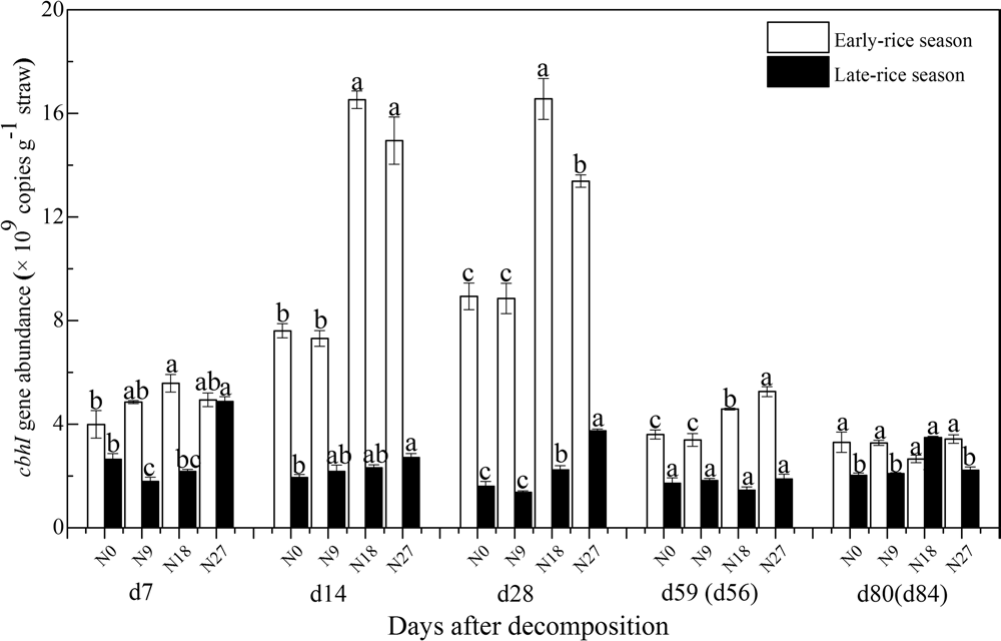
c*bhI* gene copy numbers associated with straw residual under different fertilizer treatments in double-rice rotation system. The vertical error bars represent standard error (s.e. = standard derivation/sqrt(n), n = 3) and lowercase letters indicate significant differences among fertilizer treatments in each season (*P* < 0.05; Fisher’s LSD test).

**Figure 7:**
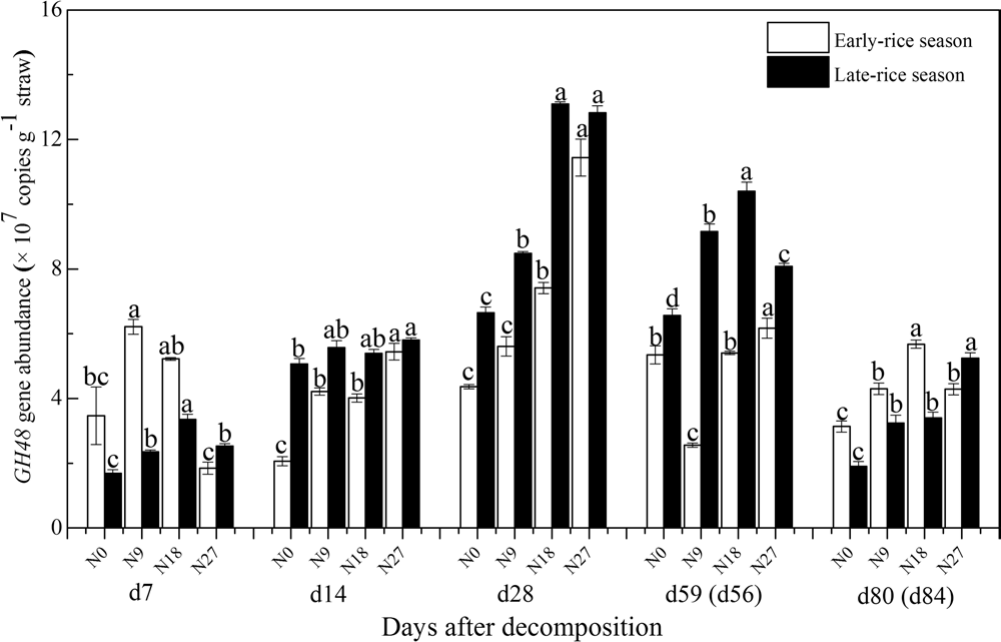
*GH48* gene copy numbers associated with straw residual under different fertilizer treatments in double-rice rotation system. The vertical error bars represent standard error (s.e. = standard derivation/sqrt(n), n = 3) and lowercase letters indicate significant differences among fertilizer treatments in each season (*P* < 0.05; Fisher’s LSD test).

Straw residues under different N fertilizer managements were used for molecular analyses. Abundance of the *cbhI* gene was in the range of 2.66–16.56 × 10^9^ copies g^–1^ straw in early-rice season and 1.37–4.88 × 10^9^ copies g^–1^ straw in late-rice season (Fig. 6). Generally, *cbhI* gene abundance in early-rice season was generally higher than that in late-rice season, and the difference was extremely significant at d14 and d28. In early-rice season, *cbhI* gene abundance increased till d28, decreased and stabilized in the following decomposition period. Meanwhile, N18 and N27 treatments generally improved straw-associated *cbhI* gene abundance at d14, d28 and d59 compared with N0 and N9 treatments. Unlike *cbhI* gene abundance in early-rice season, it was relatively stable in late-rice season with significantly highest *cbhI* gene abundance under N18 or N27 treatments except at d56. *GH48* gene abundance was approximately one of hundreds of *cbhI* gene abundance which was in the range of 1.84–11.44 × 10^7^ copies g^−1^ straw in early-rice season and 1.69–13.09 × 10^7^ copies g^−1^ straw in late-rice season (Fig. 7). *GH48* gene abundance, which was higher in late-rice season than that in early-rice season at d14, d28 and d56, increased till d28 and decreased afterwards. Approximately, the improvement of N18 and N27 on *GH48* gene abundance became more obvious along with straw decomposition process except at d7 in early-rice season. RDA analysis indicated that it was C, N, C/N ratio, P and L-leucine aminopeptidase activity that largely affected straw-associated cellulolytic gene abundance in this double-rice cropping system (Supplementary Figure S5).

## Discussion

China accounted for about 20% of the world’s rice planting area, and annual double-rice rotation system even accounted for 15% of rice planting area in China^15^. The management of crop residues has become an important aspect of sustaining long-term fertility in cropping systems. Incorporation of crop residues can change soil microbial processes, which affect nutrient availability and crop yield^16^. Thus, evaluation of straw decomposition pattern and the microbial communities associated with its decomposition under application of different N fertilizer rates could provide insights of the scientific management of crop residues.

In this study, even though crop type was the same in two seasons, we still obtained obvious differences in decomposition rate, residual straw C/N ratio and P release characteristics in different cropping seasons, which might be largely due to the different environmental factors like temperature, moisture as revealed by ABT analysis (Supplementary Table S1). To be more specific, straw decomposition rate under N18 treatment was the lowest in early-rice season, but it was the N27 treatment that showed the lowest decomposition rate in late-rice season, which also indicated that N fertilizer addition at high amounts (180 or 270 kg N ha^−1^) could strengthen the aftereffect of returning straw (Table 1)^17^. However, the differences mentioned above were not significant. We assumed soil N availability was sufficiently high for rice straw decomposition without N addition. Another possible reason for the lack of effect by N addition rate is that the C/N ratio of the rice straw was about 35 which was not particularly high. C/N might be better for forecasting the decomposition process of straw, reflecting the ratio of carbohydrates and proteins^18^. As reported, the input of straw provided C for soil microbial growth, thus increasing microbial N demand^19^. Therefore, extra N fertilizer application should provide N for microorganisms, thus restricting the litter decomposition and its N release^5^. Then we are not surprised to observe increasing C/N ratio of straw residue in the initial two weeks in both seasons, because of the relatively higher releasing speed of C than N nutrient originated from straw (Fig. 1c and d). Differences in nutrient dynamics existed among different nutrients determined and also fertilizer treatments (Supplementary Figure S2). Basically, K from returned straw was almost released in the first week, while C, N, P showed distinct release rate, suggesting that straw quality and characteristics of particular nutrient affected nutrient release or immobilization^20^.

Enzyme activities associated with straw residues represent key biological processes that link straw quality (e.g. relative availability of C and N) with the ability of microbes to assimilate nutrients for their own metabolism^21^. Thus, shifts in C- or N-acquiring enzyme activities may be linked to changes in the availability and/or storage of C and N along with straw decomposition process^22^. It is generally accepted that during the early stage of decomposition, easily available compounds are decomposed, leading to a relative increase in more recalcitrant compounds in the late decomposition stage and these changes in substrate availability are associated with changes in microbial community composition^23^. Generally, water-soluble compounds and easily decomposed materials (sugars and starch) in the residues appear to be particularly important for the high microbial activity in the initial phase which was the suitable substrate for N-acetyl-glucosamidase and L-leucine aminopeptidase activities (Supplementary Figure S3g–j)^24^. Thus, it is not surprised to see higher activities of these enzymes in the initial decomposition phase which decreased with the depletion of substrate in the late stage^5^. Previously, we expected that extra N fertilizer addition to soils would contribute the reduced C *vs*. N availability for microbial growth, thus simulating the production of C- rather N-acquiring enzymes as previously reported that increased inorganic N availability has negative effects on the activity of N-acquiring enzymes^22^. However, our investigation showed opposite results with clearly enhanced straw-associated N-acetyl-glucosamidase and L-leucine aminopeptidase activities under N18 and N27 treatment with a certain variation in both seasons (Supplementary Figure S3g–j), which probably because that inorganic N addition slightly reduced the rate of straw-N components release and thus accelerated the ability of microbes to produce enzymes for their own metabolism and further straw decomposition^25^. Another reason might be that inorganic N addition provided enough N for growth of microorganism under high C input situation, thus improving the microbial activities^5^.

After the depletion of the water-soluble materials, the more resistant fractions such as cellulose, hemicellulose, lignin and other macromolecules start to accumulate, whose decomposition requires production and release of enzymes and is therefore a more energy demanding process^23^. The decomposition of cellulose is mainly accomplished through the synergistic activities of several groups of enzymes. Enzyme β-cellobiohydrolase which cleaves cellulose into smaller oligosaccharides and β-glucosidase which finally cleaves cellobiose into its glucose constituents, play key roles in the late stage of straw decomposition^26,27^. β-xylosidase plays an important role in xylan degradation, hydrolyzing xylobiose and xylooligosaccharides to xylose from the non-reducing end^28^. Our results indicated higher activities of these three C-related enzymes in the late stage of decomposition (Supplementary Figure S3a–f). Even though the N fertilizer rate had little effect on straw decomposition rate, it was a key factor resulting in the variation of hydrolytic enzyme activities (Supplementary Table S2). Bowles *et al*.^29^ found that potential activities of C-cycling enzymes increased with inorganic N availability, which was supported by our experiment, especially under the N18 and N27 treatments (Supplementary Figure S3a–f). Cusack *et al*.^9^ found that N fertilizer addition increased the activities of some hydrolytic enzymes, but decreased oxidative enzyme activities in two tropical forests. In our study, apart from peroxidase activity in the early-rice season, both phenol oxidase and peroxidase activities showed comparable or even suppressed activities under elevated inorganic N addition (Supplementary Figure S3k–n). In contrast with hydrolytic enzymes, soil phenol oxidase has been proposed as an ‘enzymatic latch’ to protect organic C as phenolic-containing organic materials in oxygen-limited ecosystems^30^. This is because low phenol oxidase activity is conducive to the accumulation of soluble phenolics and inhibits the activity of hydrolytic enzymes, and thus benefits C sequestration^31^.

In response to the changes of residual chemistry, the microbial community composition changes as straw decomposition^32^, because microbial species differ in metabolic capacity and therefore decompose a given compound at different rates^33^. Consequently, species that can decompose a given compound more rapidly than others will become dominant in the community. It has been shown that bacteria especially dominate in the initial phases of decomposition because they can grow rapidly on easily available compounds that are present in freshly added plant residues^7,34^. Fungi grow more slowly than bacteria but can decompose more recalcitrant material and dominate in the late stage of straw decomposition^34^. Our study confirmed the ability of bacteria to colonize residues and consume labile organic C in the first step of the decomposition process. Moreover, G+ bacteria formed the largest proportions of microbial communities associated with straw during the decomposition period (Figs 2 and 3), thus confirming what was reported in a paddy soil microcosm by Kimura *et al*.^35^. A recent study suggested that in early stage of straw decomposition, the initial straw quality controls the decomposition rate and microbial community composition, whereas in the late stage of straw decomposition, the composition of the microbial community plays a greater role in controlling the straw decomposition rate^36^. At the harvest time of late-rice season, the increase in fungal PLFAs abundance coincided with a decrease in bacterial PLFA abundance, probably because many fungi were unable to compete with rapid bacterial growth and therefore increase in abundance only when the growth rate of bacteria decreases as easily available compounds become depleted^34^ or under inhibition of bacterial growth by antibiotics^37^. This trend was even clearer when we calculated the ratio of fungi/bacteria (Supplementary Figure S4d), which stayed relatively stable from d14 to d56 but significantly increased to 0.30 under the N18 treatment at d84. Furthermore, G+/G− ratio under N18 and N27 declined along with the decomposition process; however, it increased under N0 and N9 treatments until d28 and subsequently declined, indicating that inorganic N fertilizer application could provide nutrients for the growth of G−, which supports the findings for decomposers of rice straw under flooded conditions in Japan^1^. In the current study, PCA divided the rice straw samples into groups based on sampling time, but there was no obvious clustering of different fertilizer treatments (Fig. 3c and d), which might suggest inorganic N fertilizer application could regulate the C/N circumstances where organisms live but not strongly enough to affect the associated microbial community structure in short term^38^. Moreover, PCA also showed the representative characteristics of microbial communities responsible for rice straw decomposition in different stages; for example, the dominance of G− in the early stage decomposition and G+ and fungi in late stage of late-rice season (Fig. 3d). It is likely that the decomposition of labile C was closely correlated with the activity rather than biomass of microbes, especially G−, since part of the microbes remained dormant^39^. Actinomycetes were generally more abundant in the middle decomposition stage because a variety of actinomycetes play an important role in degrading lignin and lignocellulose through the excretion of peroxidases and oxidases^40^.

However, dynamics of PLFA indices in early-rice season were different from those in late-rice season. PCA results showed the representative characteristics of microbial communities responsible for rice straw decomposition were fungi in initial stage and G− in late stage (Fig. 3a and b), thus resulting in the higher fungi/bacteria ratios at d28 of early-rice season. Furthermore, G+/G− ratios under different fertilizer treatments remained low till d14, which was largely because of the relatively lower G+ PLFA abundance as a result of the competition with fungi^41^. This might correspond to the considerably lower AWCD values from d0 to d28 in the early-rice season indicated by CLPP, which focused on the functional capability of bacteria (Fig. 4a). The CLPP analysis revealed the straw-associated microbial community had a strong relationship with straw chemistry dynamics with clear variation in two seasons. As expected, easily utilized carbohydrates were the preferential substrate for straw decomposers in both seasons, followed by amines (Fig. 5a and b). Even though the AWCD values remained relatively higher in the late-rice season, inorganic N fertilizer addition could improve the metabolic ability of straw-associated decomposers in the late stage, when the main substrates were carboxylic acids (Figs 4 and 5). Compared with bacteria, multicellular actinomycetes and fungi are better known as efficient cellulose decomposers. *cbhI* and *GH48* gene as the biomarkers for cellulolytic fungi and actinobacteria were generally considered to catalyze the rate-limiting step of cellulose decomposition^42^. We observed the enhancement of their abundance under higher N fertilizer addition (180 and 270 kg N ha^−1^) and reached the highest peak shortly ahead of enzyme activities (Figs 6 and 7). RDA analysis showed that C, N, P and C/N ratio of straw residues were significantly correlated with *cbhI* and *GH48* gene abundance (Supplementary Figure S5). These results were consistent with previous reports that C/N ratio was thought to influence litter C availability, and high C/N ratio had a negative influence on extracellular hydrolytic enzymes during litter decomposition^43,44^. The characteristics of straw decomposition in agricultural soils determined by various environmental factors including climate conditions (e.g., temperature and precipitation), biotic and abiotic properties of the field (e.g. pH and contents of water, minerals, and nutrients) and tillage^45^ still need further research.

## Materials and Methods

### Site description and experimental setup

The experiment was carried out in a paddy field at Jinxian county (28°15′30″N,116°20′24′′E), Jiangxi Province, China in 2016, where double-season rice rotation is the typical cropping system. The site was located in the mid-subtropical marine monsoon climate zone with an annual average temperature and precipitation of 17.6 °C and 1790 mm, respectively. The experimental paddy soil with a clay loam texture belongs to Kandiudult (USDA soil classification). At the beginning of the experiment, the soil had a pH (H_2_O) of 5.5, 17.8 g kg^−1^ organic C, 1.7 g kg^−1^ total N, 0.6 g kg^−1^ total P, 16.8 g kg^−1^ total potassium (K)and 21.6 and 109.9 mg kg^−1^ of available P and K, respectively. Treatments of four N fertilizer rates of applications included (1) N0: no N addition, (2) N9: 90 kg N ha^−1^, (3) N18: 180 kg N ha^−1^ and (4) N27: 270 kg N ha^−1^ as urea with 75 kg P_2_O_5_ ha^−1^, 150 kg K_2_O ha^−1^ and 6000 kg ha^−1^ rice straw in each treatment. The straw decomposition experiment was based on the litter bag method. First, fresh rice straw was cut into 5-cm length. After being dried at 60 °C for 24 h, converted weight of rice straw was placed into nylon mesh bag (15 cm in length and 10 cm in width). The mesh size of the bags was 0.048 μm, which allowed free access of microorganisms (i.e. bacteria and fungi) from the soil. Bags were separately buried in a vertical position at an average depth of 15 cm.

Four bags were collected from each replicate of fertilizer treatment at 7, 14, 28, 45, 59, 73, 80 days in early-rice season and 7, 14, 28, 42, 56, 70, 84 days in late-rice season, respectively. Straw bags were transported to the lab where the exterior of the bags were brushed free of adhering soil. Roots, soil, invertebrates and frass were removed and the remaining straw weighed. Dried subsample was used for measurement and calculation of nutrient release. Subsamples of straw were immediately stored at 4 °C for enzyme activity determination and CLPP analysis and stored at −80 °C for PLFA analysis.

### Chemical and microbiological analysis of residual straw

C and N contents of initial straw and straw residues collected later from straw bags were determined by combustion on elemental analyzer (Elementar Analysensysteme GmbH, Hanau, Germany). Straw residues were digested with H_2_SO_4_-H_2_O_2_, then P and K concentration were measured using the Kjeldahl method, vanadomolybdate yellow color method, and flame spectrophotometers, respectively^46^.

A modified procedure was used to extract the microbial phospholipid fatty acid (PLFA) from 0.6 g of frozen-dried rice straw^11^. Briefly, raw lipids were extracted with a mixed solution of methanol, chloroform and citric acid (2:1:0.8) twice. Then, the glycolipid and neutral lipid fractions then were removed via passage through silicic acid-bonded solid-phase extraction columns. The resulting phospholipids were saponified and methylated to fatty acid methyl esters (FAME), which were subsequently analyzed using the MIDI Sherlock microbial identification system (Version 4.5, MIDI, Inc., Newark, DE) with FAME 19:0 as the internal standard^47^. Concentrations of PLFAs were expressed in units of nmol g^−1^. Total microbial biomass was estimated using the total concentration of PLFAs (nmol g^−1^). The abundance of individual PLFAs was indicated by their relative abundance (% mol) in each sample. The specific PLFAs that used to represent various taxonomic microbial groups biomarker in this research referenced to Zhang *et al*.^47^.

Community-level physiological profiles (CLPP) of straw-associated bacteria were evaluated using Biolog EcoPlate system (Biolog Inc., Hayward, CA), which tests the ability of bacterial community to estimate the functional diversity as previously described^11^. Mashed straw residues (0.1 g dry weight) were suspended into 49.9 ml sterile physiological saline. After shaking at 150 rpm for 30 min, the supernatant was diluted 2 times to 10^−3^ level, and 150 μl was inoculated into each well of the Biolog EcoPlates. The plates were then incubated at 25 °C for 7 days, and absorbance at 590 nm was measured every 24 h. Data at 72 h showed the largest difference among treatments and thus were used for the calculation of average well color development (AWCD) and diversity indices^48^.

Straw DNA was extracted from 0.45 g fresh straw residual using the FastDNA^®^ SPIN Kit (MP Biomedicals, Illkirch, France) and a Fast Prep-24 Homogenization System (MP Biomedicals, Irvine, CA) according to the manufacturer’s instructions. Successful DNA extraction was characterized by electrophoresis on 1% (wt/vol) agarose gels. The quantity and quality of DNA were checked using Nanodrop spectrophotometer (Nanodrop, PeqLab, Germany). Fungal glycoside hydrolase family 7 cellobiohydrolase I gene (*cbhI*) and bacterial glycoside hydrolase family 48 (*GH48*) were selected as biomarkers of cellulolytic fungi and actinobacteria, respectively. All qPCR assays were carried out in an iCycler system (BioRad, USA) using SYBR Green I chemistry and the data was analyzed by Bio-Rad iQ5 v2.0 as described previously^49^. The primers for qPCR and cloning of *cbhI* and *GH48* also the amplification protocols were listed in Supplementary Table S4. Each 20 μl reaction cocktail of qPCR contained 10 μl of 2 × SuperMix (Bio-Rad, USA), 1.6 μl of 10-fold diluted DNA, 0.4 μl each primer (10 μM) and 7.6 μl sterilized water. The amplification specificity of *cbhI* and *GH48* was confirmed by generating a melting curve, respectively. Standard curves ranging from 10^2^ to10^8^ copies were prepared by 10-fold serial dilution of known copy numbers of plasmid DNA possessing the genes of interest. qPCR was performed in triplicate and amplification efficiencies of 91.9%-96.3% were obtained with *r*^2^ values > 0.98 for *cbhI* and 87.1%-85.2% with *r*^2^ values > 0.98 for *GH48*.

### Data analysis

In order to compare litter decomposition rates under different fertilizer treatments in relation to N fertilizer rate. Decomposition rate constants (*k*) for each treatment were determined using a negative exponential model:

$${{\rm{X}}}_{t}={{\rm{X}}}_{0}{{\rm{e}}}^{\mbox{--}kt}$$

After natural log transformation, the exponential model become a general linear model. The parameters of X_0_ and *k* can be derived from the general linear model method, where *X*_*t*_ equals the amount of straw mass left at time = *t*, *X*_0_ is the initial mass of litter, and *t* = time in years^14^.

For analysis of the Biolog EcoPlate, the commonly used McIntosh’s (*U*), Shannon’s (*H*) and Simpson’s (*D*) indices were employed to assess bacterial diversity, calculated using the equations *U* = √∑*n*_*i*_^2^, *H* = −∑*P*_*i*_ (ln*P*_*i*_) and *D* = ∑ [*n*_*i*_ (*n*_*i*_ − 1)]/[*N* (*N* − 1)], respectively, where *P*_*i*_ is the proportional color development of the *i*th well (*n*_*i*_) relative to the total color development (*N*) of all wells^11,50^.

Statistical procedures ANOVA and principal component analysis (PCA) were performed with SAS and CANOCO 5 (Ithaca, NY), respectively. Other complemental calculations were performed using Origin 8, Adobe Illustrator CS4, and MS Excel 2016. Aggregated boosted tree (ABT) analysis, performed with gbmplus package in R (version 2.3), was used to evaluate the relative influence of environmental variables and numerical response variables (enzyme activity, PLFAs and CLPP). The analysis was based on the assumption that the observed variation can be fully explained by the combined effects of tested environmental variables, and the contribution of each can then be quantitatively estimated.

### Data availabilit**y**

A supplement data of this article was represented in Supplemental Material which is available online. The original datasets generated during and/or analyzed during the current study are available from the corresponding author on reasonable request.

## Electronic supplementary material

Supplenmentary materials
