# Supplementary material for: Nitrogen enrichment regulates straw decomposition and its associated microbial community in a double-rice cropping system
Source: Sci Rep. 2018 Jan 30;8:1847. doi: 10.1038/s41598-018-20293-5 (PMC5789828; doi:10.1038/s41598-018-20293-5)
Supplement: Supplementary file 1 — Supplenmentary materials [file 41598_2018_20293_MOESM1_ESM.pdf]

Title: Nitrogen enrichment regulates straw decomposition and its associated microbial community in a double-rice cropping system

Authors: Tengfei Guo, Qian Zhang, Chao Ai, Guoqing Liang, Ping He, Wei Zhou*

Ministry of Agriculture Key Laboratory of Plant Nutrition and Fertilizer, Institute of Agricultural Resources and Regional Planning, Chinese Academy of Agricultural Sciences, Zhongguancun No.12, Beijing 100081, PR China.

* Corresponding authors: Institute of Agricultural Resources and Regional Planning,

Chinese Academy of Agricultural Sciences, Beijing 100081, PR China.

Postal address: No.12, Zhongguancun South Street, Beijing.

Tel.: +8610 82108671; fax: +86 10 82106225

E-mail address: wzhou@caas.ac.cn

Supplemental Materials

2. Material and Methods

The hydrolase activities of straw residue tested including β-glucosidase, β-cellobiohydrolase, β-xylosidase, N-acetyl-glucosaminidase and L-leucine aminopeptidase were measured using 4-methylumbel-liferyl (MUB)-linked model substrates yielding the highly fluorescent cleavage products MUB on hydrolysis [1]. Specifically, each equivalent of 0.3 g dry mass of straw residue was added into centrifuge tube and was homogenized with 50 mL of 50 mM acetate buffer using a polytron homogenizer, then the mixture was poured into a round wide-mouth beaker. A magnetic stirrer was used to maintain a uniform suspension. The buffer, sample suspension, 10 mM references, and 200 mM substrates were dispensed into the wells of a black 96-well microplate according to the strict volume and order described by DeForest (2009) [1]. The microplates were covered and incubated in the dark at 25 ℃ for 4 h and the fluorescence quantified using a microplate fluorimeter (Scientific Fluoroskan Ascent FL, Thermo Fischer Scientific, Waltham, MA) with 365 nm excitation and 450 nm emission filters. The activities were expressed in unit of nmol h^{-1} g^{-1}. Activities of phenol oxidase and peroxidase were measured spectrophotometrically using the substrate of L-3,4-dihydroxyphenylalanine (L-DOPA). The dispensed volume and the order of buffer, sample suspension, 25 mM L-DOPA, and 0.3% (w/v) H_2O_2 were the same as for the fluorometric enzymes. The microplates were covered and incubated in the dark at 25 ℃ for 20 h, and the activities were assayed by measuring the absorbance at 450 nm and expressed in unit of μmol h^{-1} g^{-1}.

Reference

DeForest, J. The influence of time storage temperature and substrate age on potential soil enzyme activity in acidic forest soils using MUB-linked substrates and L-DOPA. *Soil Biol Biochem*. **41**, 1180–1186 (2009)

Table S1 Percentage contribution of environmental variables to the parameters of straw decomposition revealed by aggregated boosted tree (ABT) analysis

Variable	Remaining content (%)						Decomposition rate (year^{-1})	50% decomposition	95% decomposition
	mass	C/N	C	N	P	K			
Season	4.62	34.07	12.04	16.64	28.56	12.54	36.64	57.28	50.7
Time sampling	87.55	38.58	79.29	47.87	60.1	66.52			
N fertilizer rate	0.26	6.57	0.64	4.06	1.69	7.19	5.98	3.96	5.5
Soil mositure	3.63	7.43	2.18	8.41	5.48	6.21	34.94	22.49	23.4
Soil temperature	3.94	13.36	5.85	23.01	4.16	7.54	22.44	16.27	20.5

Table S2 Percent distribution of environmental variables to the variations of enzyme activities as revealed by aggregated boosted tree (ABT) analysis. Abbreviation: βG, β-glucosidase; βCB, β-cellobiohydrolase; NAG, N-acetyl-glucosaminidase; βX, β-xylosidase; αG, α-glucosidase; LAP, L-leucine aminopeptidase; PhOx, phenol oxidase; Perox, peroxidase.

Variable	βG	βCB	NAG	βX	LAP	Phox	Perox
Season	11.35	1.33	5.37	2.45	10.4	3.41	0.98
Time sampling	44.65	57.52	47.06	50.03	47.52	72.42	54.25
N fertilizer rate	25.84	18.01	15.76	30.48	18.68	3.64	15.01
Soil moisture	5.28	7.27	14.83	5.75	8.63	6.65	6.05
Soil temperature	12.88	15.87	16.98	11.3	14.78	13.88	23.71

Table S3 Percent distribution of environmental variables to the variations of microbial communities as revealed by aggregated boosted tree (ABT) analysis.

Variable	% variation in microbial abundance (PLFA)			% variation in metabolic diversity (CLPP)			
	Total mass	G+/G-	Fungi/Bacteria	AWCD	Mclntosh index	Shannon index	Simpson index
Season	10.90	15.36	23.04	36.07	33.71	21.3	26.49
Time sampling	25.50	44.27	31.51	44.31	46.31	29.6	30.35
N fertilizer rate	15.00	4.07	3.71	4.95	4.53	10.1	9.26
Soil moisture	23.10	17.76	19.81	4.89	6.12	14.2	17.12
Soil temperature	25.60	18.54	21.93	9.78	9.32	24.8	16.79

Table S4 Primers used for the real-time Polymerase Chain Reaction (PCR) quantification of genes for *cbhI* and *GH48* microbial community.

Primer Name	Primer sequence (5'-3')	Target gene	Thermal Profile
fungcbhIF	ACCAAYTGCTAYACIRGYAA	*cbhI*	95 ℃, 5min; 40 ×(94 ℃, 30s; 48 ℃, 45s; 72 ℃, 90s; data collection at 84 ℃ for 10s);
fungcbhIR	GCYTCCCAIATRTCCATC		
GH48_F8	GCCADGHTBGGCGACTACCT	*GH48*	94 ℃, 4min; 40 ×(94 ℃, 45s; 57 ℃, 30s; 72 ℃, 60s; data collection at 81 ℃ for 10s)
GH48_R5	CGCCCCABGMSWWGTACCA		

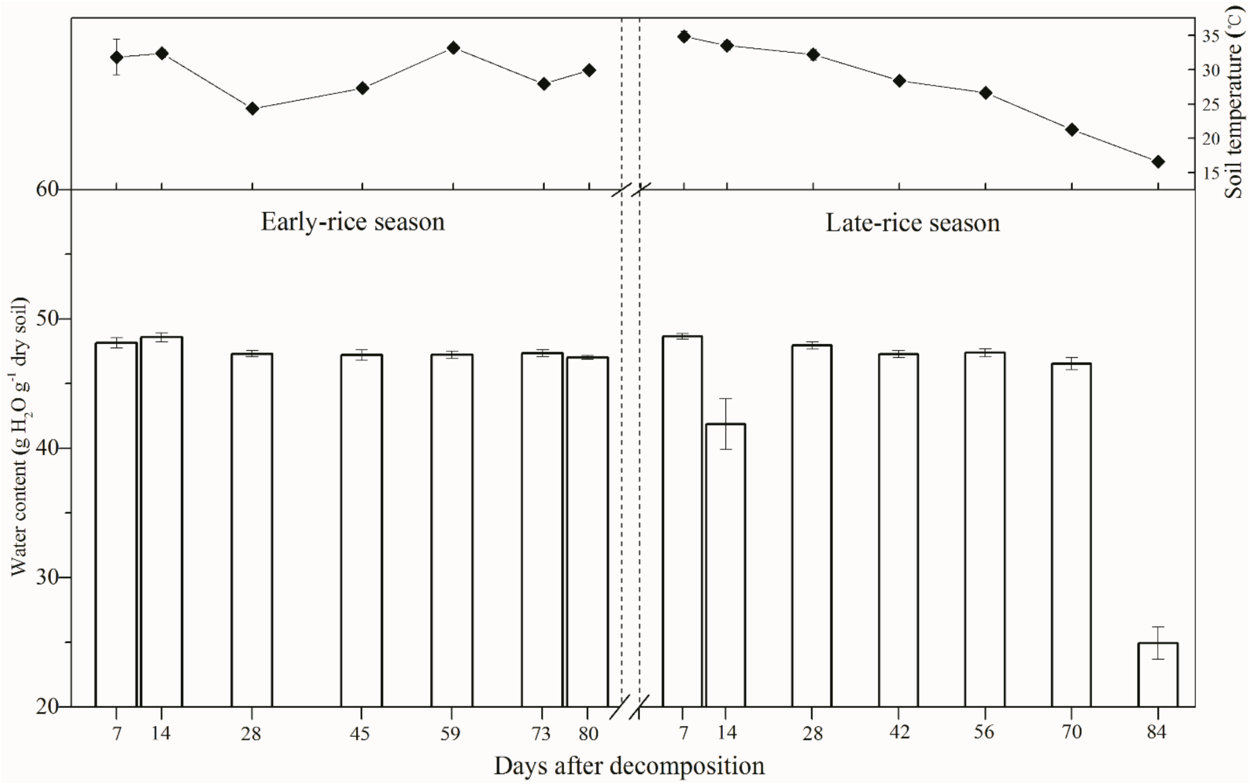

Figure S1 Soil moisture and temperature dynamics in this double-rice rotation system.

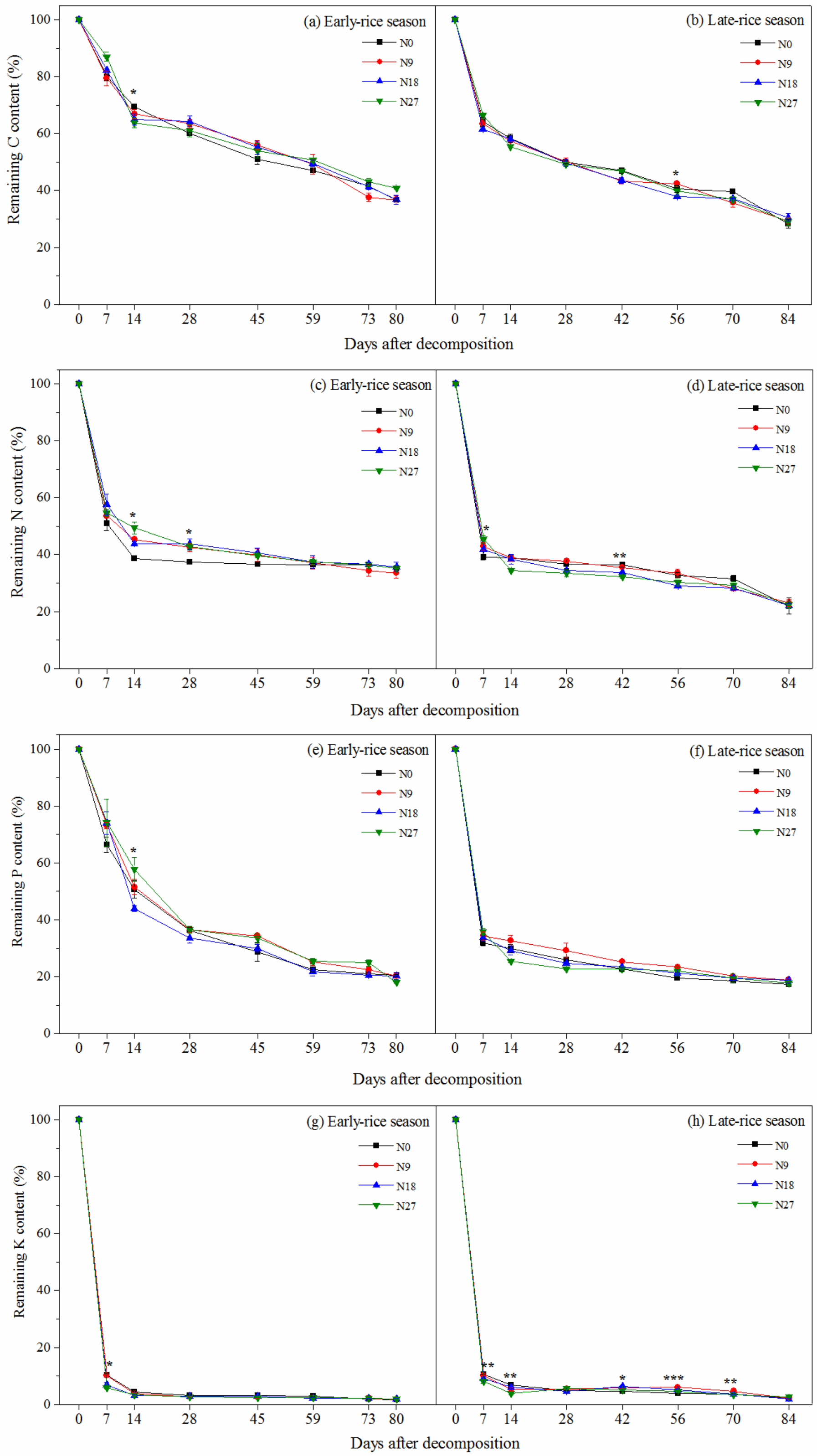

Figure S2 The cumulative loss rate (in percentage) of straw C, N, P and K under different fertilizer treatments during decomposition process in early-rice season and late-rice season.

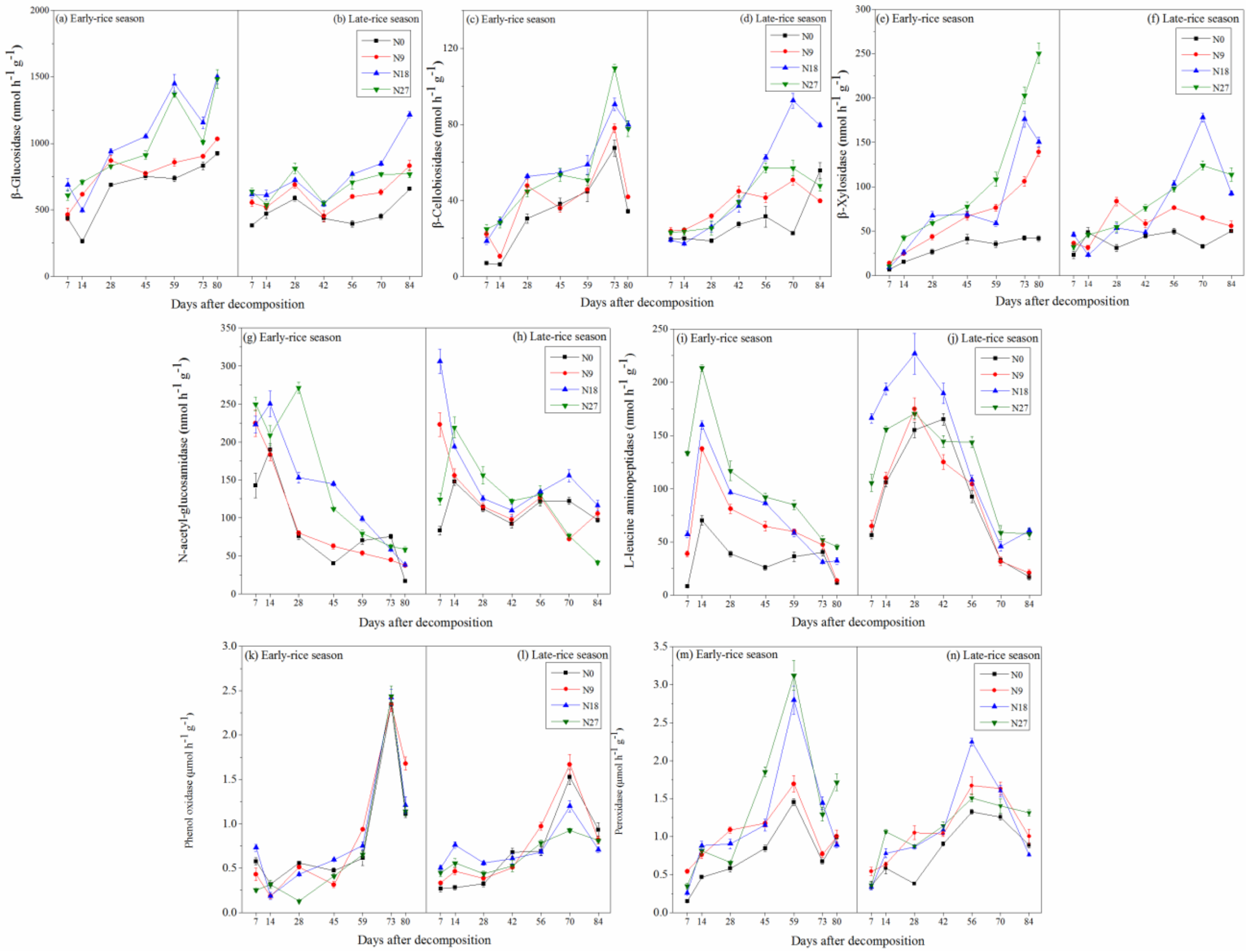

Figure S3 Straw-associated enzyme activities involved in C and N cycling under different fertilizer treatments during decomposition process in the early-rice andlate-rice seasons.

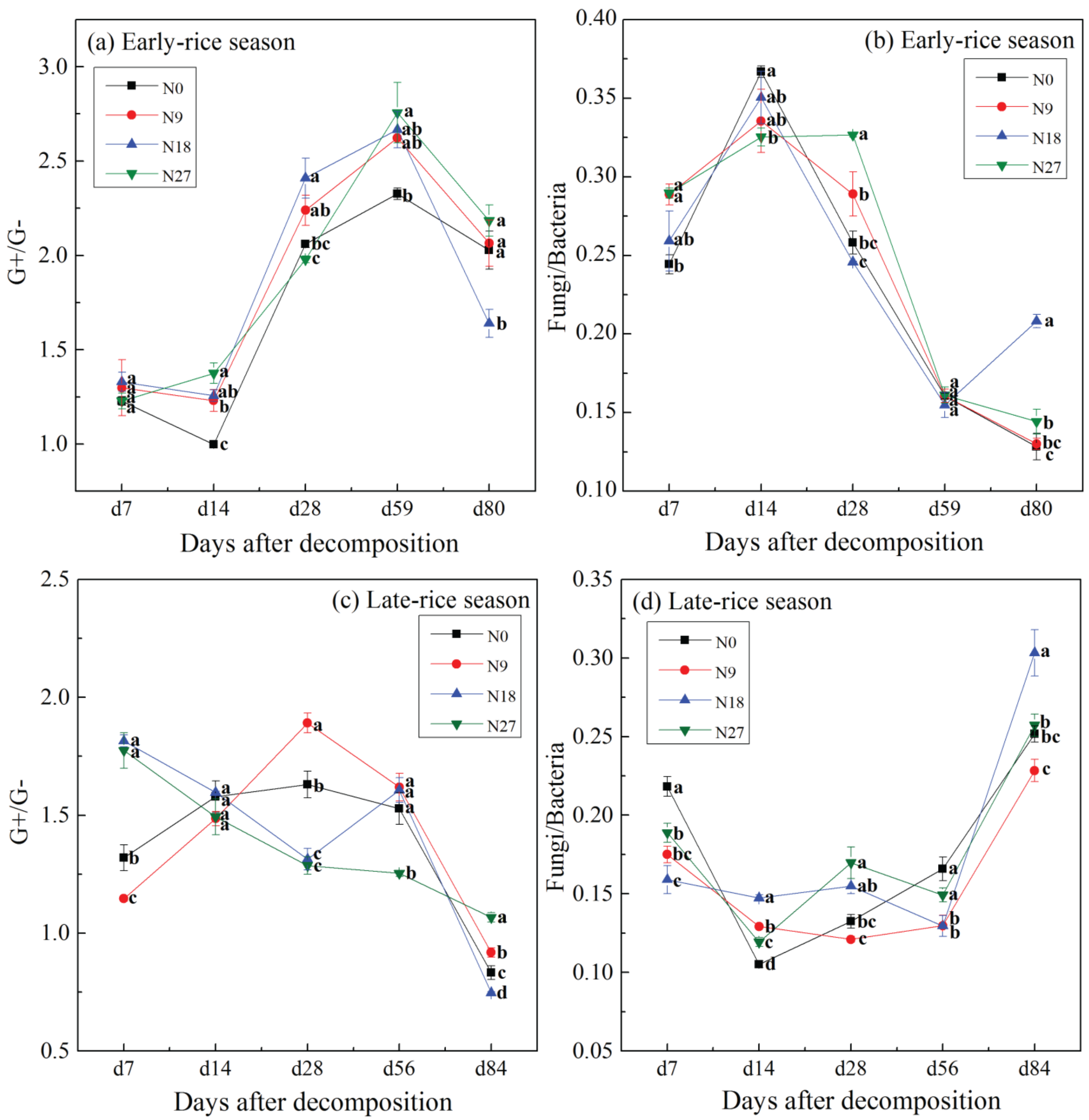

Figure S4 Dynamic comparisons of G+/G− ratio (a. early-rice season, c. late-rice season) and fungi/bacteria ratio (b. early-rice season, d. late-rice season) associated with decomposing straw under different N fertilizer addition rates as measured by PLFA analysis. Vertical bars represent the standard error (n = 3) and lowercase letters indicate significant differences among fertilizer treatments (P< 0.05).

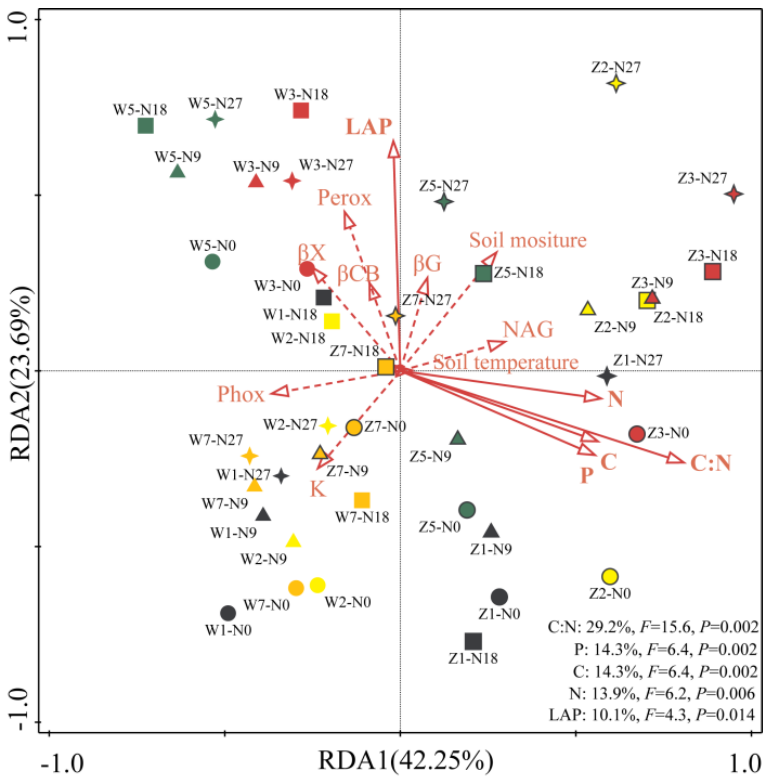

Figure S5 Redundancy analyses (RDA) of the correlations between nutrient of straw residue, straw-associated enzyme activities with cellulolytic gene abundances (*cbhI* and *GH48*). The red arrows indicate the parameters that had strong and significant impact on enzyme activities ($P < 0.05$), and corresponding explained proportion of variability was shown in the lower right corner. Z1-Z7 and W1-W7 referred to the samplingtime as d7, d14, d28, d56 and d80 (d84) in early-rice season and late-rice season, respectively.
